# What do Eulerian and Hamiltonian cycles have to do with genome assembly?

**DOI:** 10.1371/journal.pcbi.1008928

**Published:** 2021-05-20

**Authors:** Paul Medvedev, Mihai Pop

**Affiliations:** 1 Department of Computer Science and Engineering, Pennsylvania State University, University Park, Pennsylvania, United States of America; 2 Department of Biochemistry and Molecular Biology, Pennsylvania State University, University Park, Pennsylvania, United States of America; 3 Center for Computational Biology and Bioinformatics, Pennsylvania State University, University Park, Pennsylvania, United States of America; 4 Department of Computer Science, University of Maryland, College Park, Maryland, United States of America; 5 Center for Bioinformatics and Computational Biology, University of Maryland, College Park, Maryland, United States of America; University of Toronto, CANADA

## Abstract

Many students are taught about genome assembly using the dichotomy between the complexity of finding Eulerian and Hamiltonian cycles (easy versus hard, respectively). This dichotomy is sometimes used to motivate the use of de Bruijn graphs in practice. In this paper, we explain that while de Bruijn graphs have indeed been very useful, the reason has nothing to do with the complexity of the Hamiltonian and Eulerian cycle problems. We give 2 arguments. The first is that a genome reconstruction is never unique and hence an algorithm for finding Eulerian or Hamiltonian cycles is not part of any assembly algorithm used in practice. The second is that even if an arbitrary genome reconstruction was desired, one could do so in linear time in both the Eulerian and Hamiltonian paradigms.

## Introduction

When you learned about genome assembly algorithms, you might have heard a story that goes something like this:

*In the overlap-layout paradigm, solving the assembly problem requires solving the Hamiltonian cycle problem in the overlap graph. This is difficult, because the Hamiltonian cycle program is NP-hard. On the other hand, if we break our reads up into k-mers, we can build the de Bruijn graph. Then, the assembly problem becomes the problem of finding an Eulerian cycle in the de Bruijn graph, which is easily solvable in linear time. Thus, by formulating the assembly problem in terms of the de Bruijn graph, we can solve the much easier Eulerian cycle problem and not have to solve the NP-hard Hamiltonian cycle problem*.

In this paper, we explain that while de Bruijn graphs have indeed been very useful, the reason has nothing to do with the complexity of the Hamiltonian and Eulerian cycle problems.

We will first define the terms necessary to understand the above story. A Hamiltonian cycle in a graph is a cycle that visits every vertex at least once, and an Eulerian cycle is a cycle that visits every edge once. In general graphs, the problem of finding a Hamiltonian cycle is NP-hard, while finding an Eulerian cycle is solvable in polynomial time. Consider a set of reads *R*. An overlap graph constructed from *R* is a directed graph where every vertex is a read, and there is an edge from *x* to *y* if and only if a suffix (of a certain minimal length, which is given as a parameter) of *x* is equal to a prefix of *y*. Under certain idealized assumptions of the input, which we will not get into here, the genome is spelled by a cycle that visits every read exactly once, i.e., a Hamiltonian cycle of the overlap graph. Alternatively, a de Bruijn graph of *R* is a directed graph with a vertex for every *k*-long substring of *R* (called a *k*-mer) and an edge for every (*k+1*)-long substring of *R*. Under certain idealized assumptions, the genome is spelled by a cycle that visits every edge of a de Bruijn graph exactly once, i.e., an Eulerian cycle.

## The answer to the question

**Fig 1:**
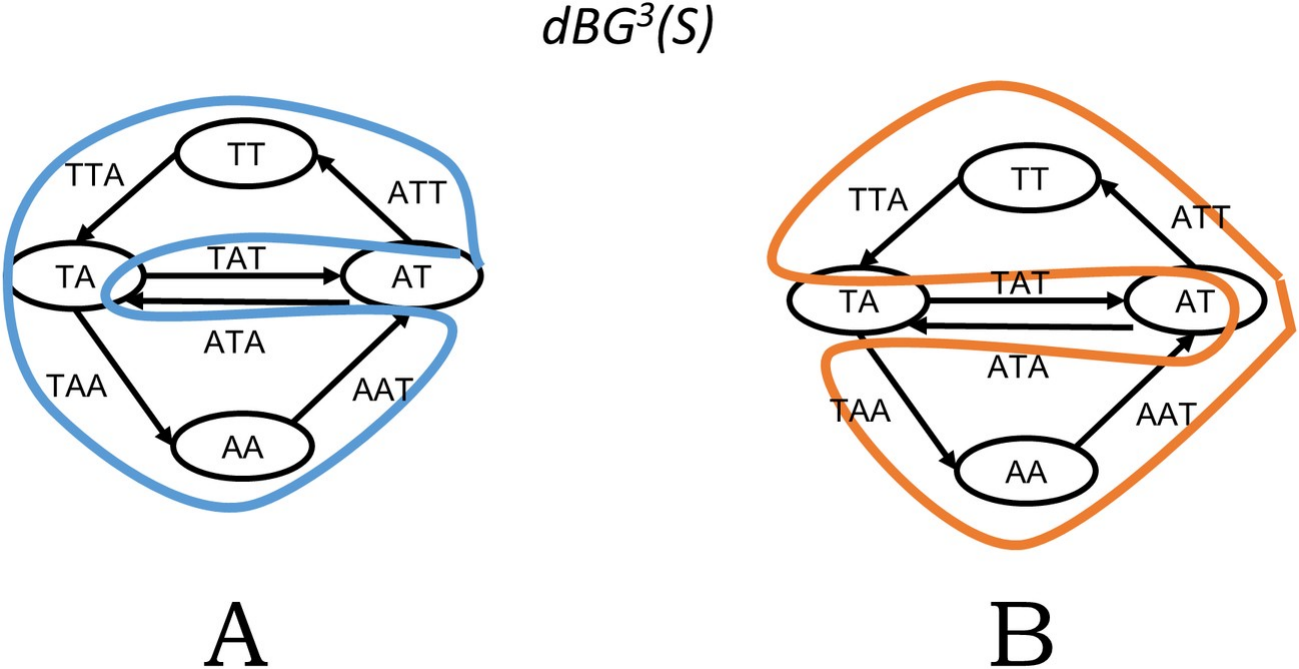
A worked out example for a set of reads *R = {TATTA*, *TAATA}* and *k = 3*. Here, the set of all k-mers is *S* = *sp*^*k*^(*R*) = {*TAT*, *ATT*, *TTA*, *TAA*, *AAT*, *ATA*}. Panel A shows *G*_1_ = *dBG*^*k*^(*S*) and one possible Eulerian cycle of *G*_1_ (in blue). Panel B show the only other Eulerian cycle in *G*_1_ (in orange). The genome reconstruction corresponding to the blue cycle is *ATTAATAT* and to the orange cycle is *ATTATAAT* (note that because the genome is circular, the last 2 characters of each string are equal to the first 2 characters).

Every Eulerian cycle in a de Bruijn graph or a Hamiltonian cycle in an overlap graph corresponds to a single genome reconstruction where all the repeats (long sequences that appear more than once) are completely resolved (i.e., their place in the genome determined). For example, Fig 1 shows 2 different Eulerian cycles in the same graph (a similar example could be constructed for Hamiltonian cycles in an overlap graph). Each cycle corresponds to a different arrangement of segments between the repeats. The presence of multiple Eulerian or Hamiltonian cycles implies that the genome structure is ambiguous given the data available. In other words, using the same set of reads, one can reconstruct different genomes, each of which is fully consistent with the data (Fig 1 gives an example). Choosing one of these reconstructions arbitrarily would be foolhardy since only one of them is the original genome. No sane assembly algorithm would do this, and that is one of the major reasons why an algorithm for finding Eulerian or Hamiltonian cycles is not part of any assembly algorithm used in practice.

Instead, assemblers output contigs—long, contiguous segments which can unambiguously be inferred to be part of the genome. Finding such segments is a very different computational problem than finding a single Eulerian or Hamiltonian cycle (see Endnote 1). In fact, it was shown that finding all possible contigs can be done in polynomial time, regardless of whether the genome reconstruction is modeled as a Hamiltonian or Eulerian cycle [1]. The algorithm used in practice (the unitig algorithm) is linear and nearly identical in the 2 graph models.

Perhaps you are not convinced by the above reasoning? Fine. For the sake of argument, let us imagine that we really are interested in finding a single, arbitrary, genome reconstruction. But even in this case, the distinction between Eulerian and Hamiltonian cycles is misleading. We make our point with this theorem, which we first state informally (a formal statement and proof will come later):

Main Theorem (informal): The following problems are equivalent and solvable in linear time:

1. Find an Eulerian cycle in the de Bruijn graph where the edges correspond to k-mers in the reads.
2. Find a Hamiltonian cycle in the de Bruijn graph where the edges correspond to all the possible (k+1)-mers that can be obtained from the reads’ k-mers.

The first part of the theorem should not be surprising. It states one half of the story we started with, namely that we can solve the assembly problem in linear time by finding an Eulerian cycle in a de Bruijn graph. The second part of the theorem, though, adds a twist. It is about finding a Hamiltonian cycle, but it differs from the initial story in 2 ways. First, it is a Hamiltonian cycle in a de Bruijn graph, not in an overlap graph. This might seem strange, but there is no special connection between overlap graphs and the Hamiltonian cycle problem—one is free to find a Hamiltonian cycle in any graph they wish. Second, the problem is solvable in linear time in this case, even though it is NP-hard in general. This might also seem strange, but in fact it is common for NP-hard problems to have polynomial time solutions for a restricted class of inputs. For example, the satisfiability problem is NP-hard in general but is polynomial time solvable when the clauses are restricted to have only 2 variables.

What the theorem states, then, is that one can solve the assembly problem in linear time by finding a Hamiltonian cycle within an appropriately defined de Bruijn graph. The fact that the Hamiltonian cycle problem is NP-hard in general graphs is not directly relevant. What is important is the underlying structure of the de Bruijn graph which makes the Hamiltonian cycle problem easy to solve (for more details, see Endnote 2). Hence, the initial story was right in the sense that using de Bruijn graphs is a good idea but wrong to imply that the complexity of the Hamiltonian cycle problem is a reason. All of this is of course assuming we are, for some reason, interested in an arbitrary genome reconstruction, which, as we argued earlier, we typically are not.

## Formal statement and proof of main theorem

We will now give some definitions to prove the main theorem. Let *t* be a string, *k* be a positive integer, and *S* be a set of *k*-mers. Let *R* be a set of reads, i.e., strings of length ≥*k*.We define *pre*_*i*_(*t*) and *suf*_*i*_(*t*) as the prefix and suffix, respectively, of length *i* of *t*. The *k*-spectrum of *R*, denoted by *sp*^*k*^(*R*), is the set of all *k*-mer substrings of the strings of *R*. The de Bruijn graph of order *k* of *S*, denoted as *dBG*^*k*^(*S*), is defined as follows. The vertex set is *sp*^*k*−1^(*S*), and for every k-mer *x*∈*S*, we add an edge from *pre*_*k*−1_(*x*) to *suf*_*k*−1_(*x*). Fig 1 shows an example of a de Bruijn graph. See Endnote 3 for more for some context about how the term de Bruijn graph is used more broadly. We define the *closure* of *S*, denoted *closure*(*S*), to be the set of all *(k+1)*-mers *y* such that *pre*_*k*_(*y*)∈*S* and *suf*_*k*_(*y*)∈*S*. Informally, it is the set of all *(k+1)*-mers that can be constructed from *S*.

The main theorem follows almost directly from definitions. The proof here is based on first principles, for expository purposes, but it is actually a corollary of deeper results (see Endnote 4).

Main Theorem (formal): Let *R* be a set of strings whose smallest length is *l*. Let *k* be a positive integer less than *l*. Then, there is a one-to-one correspondence between Eulerian cycles in *dBG*^*k*^(*sp*^*k*^(*R*)) and Hamiltonian cycles in *dbG*^*k*+1^(*closure*(*sp*^*k*^(*R*))). Moreover, an Eulerian or Hamiltonian cycle can be found in its respective graph in *O*(|*sp*^*k*^(*R*)|) time.

Proof: Let *S* = *sp*^*k*^(*R*). Let *G*_1_ = *dBG*^*k*^(*S*) and *G*_2_ = *dBG*^*k*+1^(*closure*(*S*)). First, we show that the vertex set of *G*_2_ is *S*, i.e., *sp*^*k*^(*closure*(*S*)) = *S*. Clearly, *sp*^*k*^(*closure*(*S*))⊆*S*, since no new *k*-mers are created during the closure process. Now, let *x* be a *k*-mer in *S*. It must appear in some read *r*, and since the length of *r* is greater than *k*, *x* must be a prefix or suffix of some *(k+1)*-mer *y* in *R*. Moreover, *y* must be in *closure*(*S*), since its prefix and suffix are both in *r* and hence in *S*. Therefore, *x*∈*sp*^*k*^(*closure*(*S*)), completing our proof that *S*⊆*sp*^*k*^(*closure*(*S*)) and the vertex set of *G*_2_ is *S*.

**Fig 2:**
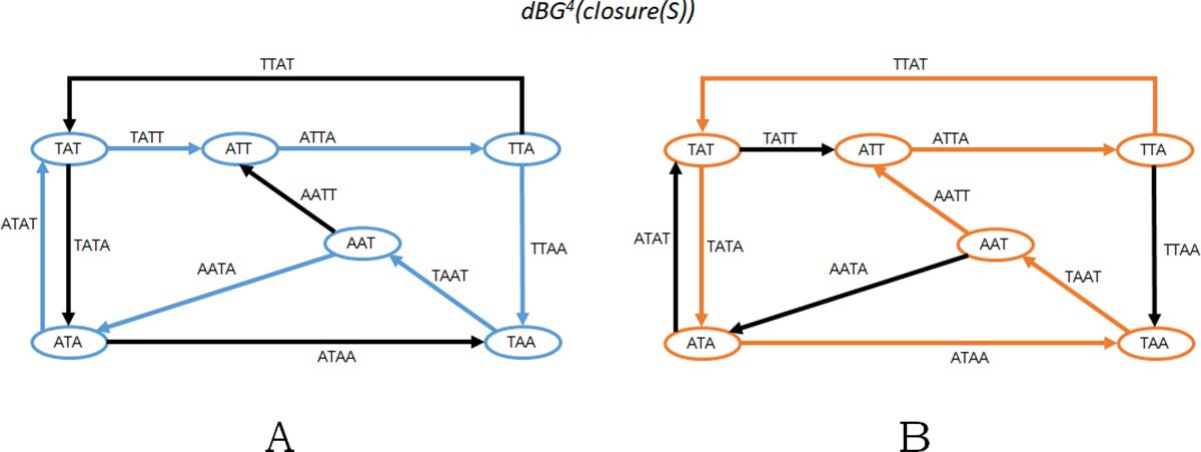
Using the same example as in Fig 1, this figure shows *G*_2_ = *dBG*^*k*+1^(*closure*(*S*)) and the 2 possible Hamiltonian cycles in *G*_2_. Notice that the *k*-mer sequence corresponding to the blue cycle in Panel A is the same as in Fig 1A; similarly, the *k*-mer sequence for the orange cycle in Panel B is the same as in Fig 1B.

Observe that a sequence of k-mers *C*_1_ = *x*_0_,…,*x*_*n*−1_ is a sequence of edges defining an Eulerian cycle in *G*_1_ if and only if the set of k-mers of *C*_1_ is exactly *S* (without any repetitions) and, for all *i*, *suf*_*k*−1_(*x*_*i*_) = *pre*_*k*−1_(*x*_*i*+1 *mod n*_). Also, observe that a sequence of k-mers *C*_2_ = *x*_0_,…,*x*_*n*−1_ is a sequence of vertices defining a Hamiltonian cycle in *G*_2_ if and only if the exact same criteria holds, i.e., the set of k-mers of *C*_2_ is exactly *S* (without repetitions) and, for all *i*, *suf*_*k*−1_(*x*_*i*_) = *pre*_*k*−1_(*x*_*i*+1 *mod n*_). Thus, there is a one-to-one correspondence between Eulerian cycles in *dBG*^*k*^(*S*) and Hamiltonian cycles in *dBG*^*k*+1^(*closure*(*S*)). Fig 2 shows an example.

For the running time, an Eulerian cycle can be found in time linear in the number of edges using a classical algorithm, e.g., Hierholzer’s Algorithm. Giving the one-to-one correspondence above, the vertex labels of a Hamiltonian cycle in *G*_2_ can be found by outputting the edge labels of an Eulerian cycle in *G*_1_. Hence, the running times for the 2 problems are equivalent.

## Conclusions

So why are de Bruijn graphs so popular for short read assembly, if not for the difference in the complexity of finding Eulerian or Hamiltonian cycles? The answer is complex, which might explain why the initial simple story was appealing. It may have to do with the simplicity of their implementation, the appeal of the k-mer abstraction, the ease of error correction, or with something else. In fact, the difference between using de Bruijn graphs and overlap graphs is poorly understood and is a fascinating open research problem. But, the Eulerian and Hamiltonian cycle dichotomy is not really relevant to assembly or to the popularity of de Bruijn graphs.

## Endnotes

1. For those who are curious, the problem of finding all possible contigs was first mentioned in [2] and later explored by [1] and other follow-up papers. In this line of research, the set of substrings which must appear in all possible genome reconstructions are called omnitigs. For a broader discussion on how to formulate the genome assembly problem, see the tutorial by Medvedev [3].
2. There is much more that has been said on the complexity of finding a single genome reconstruction. For a starting point, see the papers [2,4–6]. The last 2 papers [5,6], especially, show how the complexity depends more on the repeat structure of the underlying genome than on the graph model used.
3. The definition of de Bruijn graph that we give here is sometimes referred to as the edge centric dBG, not to be confused with a node centric one (see https://www.biostars.org/p/175058/#256741 for an explanation of the 2 terms). Historically, the term “de Bruijn graph” had a different definition, which is still used today in other contexts, especially outside of bioinformatics. There, the "full" de Bruijn graph of order *k* is not defined with respect to a set of *k*-mers *S*, but, instead, has the edge set as the universe of all *k*-mers [7,8]. The de Bruijn graph as defined here is then the subgraph induced by the set of edges *S* in the full de Bruijn graph.
4. The main theorem can be viewed as a corollary of a similar result for full de Bruijn graphs (see Endnote 3) and of the relationship between Eulerian cycles in a digraph *G* and Hamiltonian cycles in the line digraph of *G*. The full de Bruijn graph of order *k+1* is the line digraph of the full de Bruijn of order *k* (Lemma 2.5.1 in [9]). Using this fact, de Bruijn already observed in his original paper that there is a one-to-one correspondence between Eulerian cycles in the full de Bruijn of order *k* and Hamiltonian cycles in the full de Bruijn graph of order *k+1* [3]. The proof in this paper can be viewed as first arguing that *G*_2_ is the line digraph of *G*_1_ and then using the same type of argument as de Bruijn [3] to show the one-to-one correspondence.
